# Editorial: Age-Related Vestibular Loss: Current Understanding and Future Research Directions

**DOI:** 10.3389/fneur.2017.00443

**Published:** 2017-08-30

**Authors:** Yuri Agrawal

**Affiliations:** ^1^Johns Hopkins University, Baltimore, MD, United States

**Keywords:** vestibular diseases, aging, vestibular function tests, falls, dizziness

**Editorial on the Research Topic**

**Age-Related Vestibular Loss: Current Understanding and Future Research Directions**

This Research Topic reflects the collective work of 44 authors from around the world yielding 11 thoughtful and provocative publications. Several themes have clearly emerged from this body of work, which help to establish where we are in understanding age-related vestibular loss and the fundamental research gaps that we must address. First and foremost, it is clear that we are dealing with a topic of tremendous public health significance. The global population is aging, and age-related degeneration of the vestibular system is a widespread phenomenon that occurs as part of the normal aging process. Older individuals disproportionately experience falls, which are a disastrous event associated with tremendous morbidity and early mortality. It is known that the vestibular system contributes to fall risk; however, the extent to which vestibular loss contributes to falls in older adults is not precisely known (and may differ across individuals). Moreover, although falls are highly common and age-related vestibular loss is widespread, vestibular therapies such as vestibular rehabilitation are seldom offered to the large number of older adults presenting with falls in the primary care setting. Much work needs to be done to provide a strong, quantitative evidence base for the causal relationship between vestibular loss and falls in older adults and the benefit of vestibular therapy.

A second theme that follows from the first is the clear need for efficient clinical tests that identify clinically meaningful vestibular loss in older adults. The vestibular system is a highly complex structure that encompasses five peripheral end-organs and widespread central connections through brainstem nuclei, the cerebellum, the thalamus, vestibular cortex, and hippocampus. The function of the vestibular system can be probed at many levels, based on anatomy (e.g., canal vs. otolith, peripheral vs. central), based on level of analysis (e.g., cellular neurotransmitters vs. cortical networks, reflex vs. perceptual testing), and based on functional behaviors (e.g., gait vs. spatial orientation). Moreover, in the context of aging, vestibular loss is typically one of the multiple concomitant deficits that may be occurring and contributing to a given clinical phenotype (e.g., dizziness, imbalance, and falls). Specifically, older adults may also have deficits in proprioception, vision, hearing, and muscle strength. Further, even if sensorimotor function is relatively intact, older adults may have deficits in central integration of these various sensory signals to generate a coherent motor output. Additionally, older individuals may compensate to varying degrees for their deficits, such that inadequacies of compensation may also contribute to the clinical picture. It is, therefore, critical for useful clinical tests to disambiguate the various layers of potential contributing factors (i.e., primary sensorimotor deficits, deficits in central integration, and deficits in compensation) within a given older adult, with the goal of providing “personalized” strategies to improve balance function.

The publications in this Issue highlight numerous vestibular assays that differentiate older from younger individuals. Chau et al. observed reduced vestibulo-ocular reflex responses to rotational stimulation among older adults with dizziness. Bermúdez Rey et al. observed increasing vestibular perceptual thresholds beginning at the age of 40, and the authors also found that higher roll tilt thresholds were associated with poorer postural stability. Chiarovano et al. reported that older adults with postural instability did not experience a rotation perception during warm caloric irrigation. They termed this phenomenon “vestibular neglect,” and suggested it arose from reduced central responsivity to peripheral stimulation. Maheu et al. provide a review of age-related differences in performance on the standard clinical vestibular tests. Several studies also considered how aging might lead to deficits in central vestibular processing. At a molecular level, Smith discussed the differences in neurotransmission occurring at the level of the vestibular nuclear complex in older vs. younger animals. Xie et al. showed poorer performance among older adults on a test of spatial navigation, the triangle completion task, relative to younger individuals. Arshad and Seemungal reviewed several recent studies that reported reduced connectivity of central vestibular networks associated with increased age. Two studies specifically evaluated whether compensation for vestibular loss differs between young and old adults. Vestibular compensation relies on central mechanisms (cerebellar, brainstem, striatal, etc.) and may thus be a measure of central nervous system rather than vestibular function. Scheltinga et al. reported that older adults with acute unilateral vestibular loss (aUVL) experienced greater balance impairments compared to younger individuals with aUVL. Moreover, the balance impairments in older individuals took longer to improve and were less likely to resolve. Anson et al. examined how age influenced the generation of compensatory saccades following horizontal head impulse testing. They observed that increased age was associated with larger compensatory saccade amplitude, even after accounting for underlying VOR gain.

These studies all assess vestibular function at a different level of analysis. However, the “holy grail” would be a test that provides a comprehensive snapshot of the extent to which the observed vestibular impairment contributes to the individual’s symptoms relative to other impairments, and the individual’s level of compensation. As stated by Fernández et al., “Reaching a complete, meaningful, and treatment-oriented diagnosis in elderly dizzy patients remains an important challenge for even the most experienced clinician.” New techniques and assays will likely be needed to accomplish this task. For example, postural assessment is becoming increasingly powerful through frequency-based analyses and analyses of complexity. It is possible that a simple postural test can determine the source of an individual’s sway (e.g., reduced proprioception vs. vestibular function) based on the sway frequencies observed. Moreover, as discussed by Anson and Jeka, assays that are more ecologically valid (e.g., accelerometry deployed during routine daily activities) may be more sensitive at detecting deficits that manifest dynamically during daily activities but that may not be evident in a clinical laboratory. Finally, assays that measure central vestibular processing and compensation may prove useful in understanding the clinical picture and also designing therapy. Currently, functional imaging studies provide insight into these processes, although other measures that can be more easily scaled may prove more useful in the clinical setting.

This research topic clearly highlights a growing awareness of age-related vestibular loss, and its potentially far-reaching impact on the public’s health. Indeed, efforts are currently underway by the Barany Society’s International Classification of Vestibular Diseases to codify diagnostic criteria for age-related vestibular loss. As discussed, age-related vestibular loss is a complex phenomenon to manage clinically and study scientifically, largely owing to the complexity of the vestibular system as well as the complexity of the aging process. This is both a challenge and an opportunity to harmonize our conceptual framework, standardize our assessment tools, and catalyze innovation and new developments in this field.

## Author Contributions

The author confirms being the sole contributor of this work and approved it for publication.

## Conflict of Interest Statement

The author declares that the research was conducted in the absence of any commercial or financial relationships that could be construed as a potential conflict of interest.

